# Reciprocity in Medical Licenses Again

**Published:** 1901-06

**Authors:** 


### RECIPROCITY IN MEDICAL LICENSES AGAIN.

WITH the oncoming of the spring meetings of the several medical bodies that group themselves around the American Medical Association, the question of reciprocity in medical licenses between the states comes up to us with its perennial wellspring of woes and wails. While it is regarded as a desirable condition to reach, we are quite strongly of the opinion that altogether too much importance is given the matter, by those who discuss it simply from a theoretical viewpoint.

It may be a matter of great convenience for the young physician who has obtained permission to practise in Arkansas, to transfer the field of his professional work to New Jersey upon a mere indorsement of his license without undergoing an examination by the board of medical examiners in the latter state. Furthermore, it may be a convenience for the professor in Missouri to remove to New York and begin the practice of medicine without let or hindrance from the laws of the Empire State. These, however, are matters of personal comfort affecting the individuals interested alone, and not at all the medical interests of the commonwealths referred to.

What more particularly concerns the state is, that the men and women licensed to practise medicine shall be amply equipped to do so. Its demands are made with a view to the protection of its citizens from ignorance, incompetency and fraud, and not from any desire to embarrass the citizens of other states who desire to come across its borders to engage in the practice of medicine. If this seems to be a part of its purpose, or if in effect it really proves embarrassing to a few who desire to change residences, this is merely a circumstance that should in nowise affect the general purposes of the practice laws. These statutes have elevated the standard of medical education throughout the United States,—in some states considerably, in others a little,—so that in the aggregate there has been great improvement. No doubt, within the next ten years the gain in this direction will be so great that very little difference will exist between the licensing standards of Arizona and Pennsylvania, or between those of Oregon and Florida; and in this way will come the solution of the reciprocity question, which seems so disturbing to a number of most excellent gentlemen who are discussing it.

We are led to these remarks at this time particularly because of an article lately sent out to the lay press by Dr. Emil Amberg, of Detroit. In his letter of transmittal he says: "Uniform medical legislation is one of the greatest questions before the people of our country at the present time." It seems to us that Dr. Amberg has exaggerated to a considerable degree the so-called importance of interstate reciprocity in medical licenses. In our view, there are a number of questions that should take priority of this one, in the minds of the people of this country at the present moment. However, we are not disposed to enter into captious criticism of the verbiage of

Dr. Amberg's message to the laity, when his intent is so unmistakably and undeniedly good. It is rather with the essence of the question, that the profession of medicine should concern itself in discussing reciprocity in licensure.

We have said before, and we repeat it now, that of far more importance is the establishment of uniform standards of medical education. In other words, equality of standards is the true basis of reciprocity. Our precise words in an address to the Confederation of State Examining Boards were: "The only equitable basis upon which reciprocity can be established, that appears both feasible and practicable, is that of equality of standards for admission to the study and practice of medicine. This implies an equalisation of the preliminary requirements of medical students and a uniformity of applying the tests; a uniform period of collegiate training, including uniformity of methods of teaching; and finally an absolute similarity in the methods of conducting state examinations and granting licenses." Doubtless this idea could be better expressed, but the general principle is as pertinent now as when uttered.

It is of less importance to medical colleges to obtain a division of examinations, than it is to improve their standards of teaching. It is of more consequence to instruct the plastic minds of pupils in a manner so substantial, that they will at least retain the knowledge so imparted long enough to meet the requirements of the state test, without dividing the examinations in order to permit examinations in chemistry, physiology, anatomy and hygiene at the end of two years. In other words, it should be the object of the colleges to teach their students medicine, and not to act as mere preparatory schools for the state examining boards.

The New York *Medical Record* has discussed the question of reciprocity on several occasions with great intelligence and forcefulness. In its issue of March 16, 1901, it states the case so clearly that he who runs may read.

